# Linking the Center for International Blood and Marrow Transplant Research Registry to the California Cancer Registry and California Hospital Patient Discharge Data

**DOI:** 10.1016/j.jtct.2022.09.016

**Published:** 2022-09-27

**Authors:** Theresa H.M. Keegan, Ann Brunson, Julianne J.P. Cooley, Sara J. Schonfeld, Christa L. Meyer, Bryan Valcarcel, Renata Abrahao, Ted Wun, Jeffery Auletta, Lori Muffly, Lindsay M. Morton

**Affiliations:** 1 Center for Oncology Hematology Outcomes Research and Training, Division of Hematology and Oncology, University of California Davis Comprehensive Cancer Center, Sacramento, California; 2 California Cancer Reporting and Epidemiologic Surveillance Program, University of California Davis Comprehensive Cancer Center, Sacramento, California; 3 Radiation Epidemiology Branch, Division of Cancer Epidemiology and Genetics, National Cancer Institute, National Institutes of Health, Department of Health and Human Services, Bethesda, Maryland; 4 Center for International Blood and Marrow Transplant Research, National Marrow Donor Program/Be The Match, Minneapolis, Minnesota; 5 Divisions of Hematology/Oncology/BMT and Infectious Diseases, Nationwide Children’s Hospital, Columbus, Ohio; 6 Division of Blood and Marrow Transplantation and Cellular Therapy, Stanford University, Stanford, California

**Keywords:** Hematopoietic cell, transplantation, Hematologic neoplasm, Cancer, Malignancy, Registry, Children, Adolescents, Adults

## Abstract

Advances in hematopoietic cell transplantation (HCT) have substantially improved patient survival, increasing the importance of studying outcomes and long-term adverse effects in the rapidly growing population of HCT survivors. Large-scale registry data from the Center for International Blood and Marrow Transplant Research (CIBMTR) are a valuable resource for studying mortality and late effects after HCT, providing detailed data reported by HCT centers on transplantation-related factors and key outcomes. This study was conducted to evaluate the robustness of CIBMTR outcome data and assess health-related outcomes and healthcare utilization among HCT recipients. We linked data from the CIBMTR for California residents with data from the population-based California Cancer Registry (CCR) and hospitalization information from the California Patient Discharge Database (PDD). In this retrospective cohort study, probabilistic and deterministic record linkage used key patient identifiers, such as Social Security number, ZIP code, sex, birth date, hematologic malignancy type and diagnosis date, and HCT type and date. Among 22,733 patients registered with the CIBMTR who underwent autologous or allogeneic HCT for hematologic malignancy between 1991 and 2016, 89.0% were matched to the CCR and/or PDD (n = 17,707 [77.9%] for both, n = 1179 [5.2%] for the CCR only, and n = 1342 [5.9%] for the PDD only). Unmatched patients were slightly more likely to have undergone a first autologous HCT than an allogeneic HCT (12.6% versus 9.0%), to have a larger number of missing linkage identifiers, and to have undergone HCT prior to 2010. Among the patients reported to the CIBMTR who matched to the CCR, 85.7% demonstrated concordance of both hematologic malignancy type and diagnosis date across data sources. This linkage presents unparalleled opportunities to advance our understanding of HCT practices and patient outcomes.

## INTRODUCTION

The application and curative potential of hematopoietic cell transplantation (HCT) for the treatment of hematologic malignancies, hereditary blood disorders, and certain autoimmune diseases has increased over the past several decades [1]. The annual number of HCTs performed in the United States has more than quadrupled since the early 1990s, with approximately 8900 allogeneic HCT recipients and 14,000 autologous HCT recipients in 2018 [2,3]. Improved survival and expansion of patient eligibility, particularly in older individuals, is predicted to result in dramatic growth of the population of US HCT survivors, from an estimated >100,000 in 2009 to 500,000 by 2030 [1,4].

The expansion of HCT and growing awareness of unique survivorship needs of recipients have increased the importance of studying outcomes and adverse effects in HCT survivors. Previous research has demonstrated that HCT recipients are at risk of developing a range of short-term and long-term complications, including subsequent malignancies, cardiovascular disease, infections, and premature mortality [5–12]. However, large numbers of patients with systematic, long-term follow-up from a variety of transplantation centers are needed to fully describe these adverse outcomes, which have been shown to vary substantially based on age and other patient demographic and clinical factors [6,8,10,13].

The largest and most comprehensive HCT registry in the United States is maintained by the Center for International Blood and Marrow Transplant Research (CIBMTR), a research collaboration between the National Marrow Donor Program/Be The Match and the Medical College of Wisconsin that systematically collects extensive HCT and outcome data for more than 600,000 patients from more than 500 transplantation centers internationally [14]. The CIBMTR holds the contract for the Stem Cell Therapeutic Outcomes Database, which was awarded in 2006 and mandates reporting of all allogeneic HCTs performed in the United States [2]. Reporting is voluntary for autologous HCTs, but an estimated 85% of these HCTs are captured [2]; however, the completeness and accuracy of HCT center-reported data, particularly for long-term outcomes, have not been systematically compared with other administrative or population-based data sources.

To evaluate the robustness of long-term outcome data in the CIBMTR registry and assess health-related outcomes and healthcare utilization among HCT recipients, we linked the CIBMTR database for California residents with the population-based California Cancer Registry (CCR) and hospitalization information from the California Department of Health Care Access and Information Patient Discharge Database (PDD) for all patients diagnosed with a hematologic malignancy between 1991 and 2016. We selected these databases for linkage because of their large scale and high data quality. Specifically, 11% of all US HCT recipients underwent transplantation in California in 2016 to 2020, the CCR has been estimated to capture >98% of all cancer diagnoses occurring in California residents, and the PDD captures all inpatient hospitalizations from nonfederal hospitals across the state [3,15,16]. In this article, we describe our linkage methodology, compare characteristics of the study populations by match status, and evaluate the concordance of HCT indications (ie, hematologic malignancy type) and HCT types among data sources.

## METHODS

### CIBMTR

Since 2007, participating CIBMTR institutions have been required to report data from all consecutive allogeneic HCT procedures on standardized data collection forms completed by data managers and submitted at the time of HCT and at 100 days, 6 months, and annually post-HCT until year 6 and biennially thereafter until death [2,14,17]. Most centers also voluntarily report autologous HCT procedures on the same forms [14]. The CIBMTR collects data at 2 levels: Transplant Essential Data (TED) and Comprehensive Report Form (CRF) levels. The TED dataset is an internationally accepted standard dataset that contains a limited number of key variables for all consecutive HCT recipients. TED-level data, with some additional details of donor and graft characteristics, comprise the obligatory data submitted to the Stem Cell Therapeutic Outcomes Database. When an HCT is registered with the CIBMTR, a subset of patients is selected for the CRF level of data collection through a weighted randomization scheme. The CRF captures additional patient-, disease-, and treatment-related data. The broad range of information reported to the CIBMTR includes patient demographics, clinical characteristics, HCT details, transplantation center, and key clinical outcomes, including relapse, acute and chronic graft-versus-host disease, common HCT complications, vital status and cause of death, and selected late effects for patients on the CRF data collection track) [14]. Data compliance and quality are monitored by onsite audits, computerized checks for discrepancies, and physicians’ review of submitted data. Studies are performed in compliance with all applicable federal regulations pertaining to the protection of human research participants. Patients and/or guardian(s) provide written informed consent for data submission and research participation.

Within the CIBMTR database, patients eligible for the linkage underwent allogeneic or autologous HCT for a hematologic malignancy (Table 1) diagnosed between January 1, 1991, and December 31, 2016, consented for research, and were California residents based on ZIP code at the time of HCT or transplantation at a California HCT center if residential ZIP code was missing. For subsequent analyses, the hematologic malignancy type that was the indication for HCT was categorized according to the World Health Organization’s Classification of Tumors of the Hematopoietic and Lymphoid Tissues, 4th Edition (Supplementary Table S1A) [18]. HCT centers were characterized by volume based on the total number of allogeneic HCTs performed at each center over the entire study period, grouped into tertiles (low, <140; medium, 140–459; high, ≥460) to investigate the relationship of HCT volume with data completeness and quality.

### CCR

The CCR is California’s population-based cancer surveillance system since 1988, collecting cancer incidence on approximately 99% of new cancer cases (www.ccrcal.org) and harmonizing data from the regional cancer registries within the state. Information is collected on patient sociodemographics (eg, age at diagnosis, sex, race/ethnicity, neighborhood socioeconomic status, health insurance at diagnosis/initial treatment), tumor characteristics (eg, date of diagnosis and tumor site, histology, and stage at diagnosis), initial treatment (eg, chemotherapy/immunotherapy, radiotherapy, surgery), and HCT (data collection required for patients diagnosed from 2003 forward) [15,19]. The CCR also obtains vital status, last known follow-up date, and underlying cause of death captured on the death certificate through hospital follow-up and linkages to state and national vital statistics and other databases. In addition, the CCR has established linkage mechanisms with LexisNexis to obtain updated address and determine whether patients have emigrated from the state.

Within the CCR, patients eligible for linkage were diagnosed with a hematologic malignancy (Table 1) between January 1, 1991, and December 31, 2016. For subsequent analyses, hematologic malignancy types recorded in the CCR were categorized according to the World Health Organization’s Classification of Tumors of the Hematopoietic and Lymphoid Tissues, 4th Edition (Supplementary Table S1B) [18].

### PDD

Since 1991, the California Department of Health Care Access and Information has mandated reporting of diagnostic and procedure codes on all inpatient hospitalization admissions in California from nonfederal hospitals across the state into the PDD (https://hcai.ca.gov/). For all hospital admissions, the PDD data provides patient demographics (eg, age, sex, race/ethnicity, ZIP code of residence, health insurance), hospital, and clinical information (eg, admission and discharge date, principal diagnosis and up to 24 secondary diagnoses, principal procedure and up to 20 secondary procedures). Diagnoses and procedures were coded based on the International Classification of Diseases, Ninth (ICD-9) and Tenth (ICD-10) Revisions.

### Linkage

**Figure 1.**
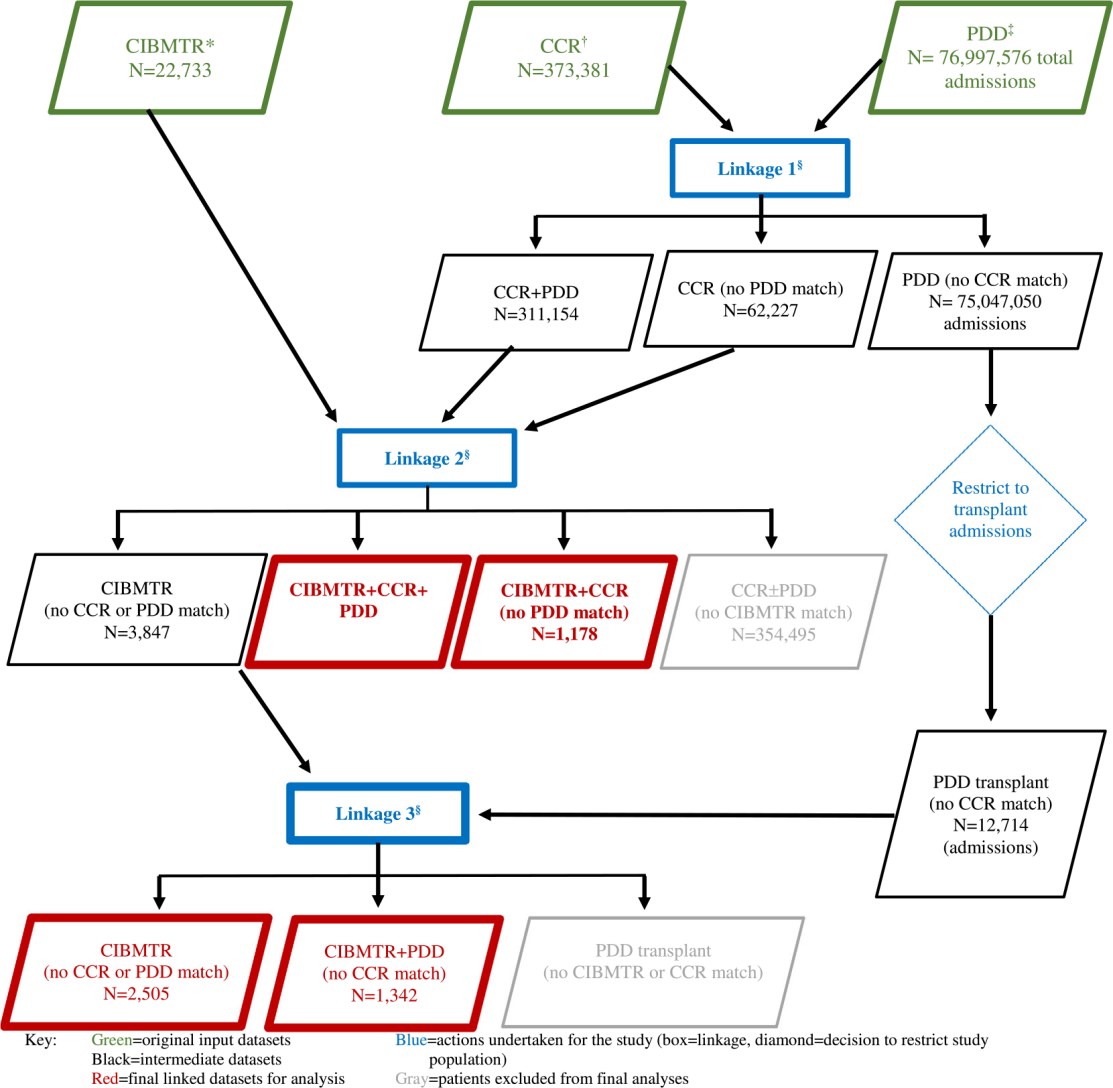
Process of linking data for patients with hematologic malignancies in the CIBMTR database to the CCR and admissions in the California PDD. *CIBMTR cohort inclusion criteria: patients who underwent allogeneic or autologous HCT for hematologic malignancy (Table 1) between 1991 and 2016, provided consent for research, and are California residents (based on ZIP code at the time of HCT or California HCT center if residential ZIP code is missing). ^†^CCR cohort inclusion criteria: all California residents diagnosed with hematologic malignancies (Table 1) between 1991 and 2016. ^‡^California PDD admission inclusion criteria: all nonfederal hospital admissions between 1991 and 2016. ^§^Linkages: see Table 2 for linkage identifiers used in each database and Table 3 for combinations of linkage identifiers used to determine a match.

The protocol for this study was approved by the Institutional Review Boards of the University of California Davis, the California Committee for the Protection of Human Subjects, and the National Marrow Donor Program and was determined to not be human subjects research by the National Cancer Institute. We undertook the linkage of the CIBMTR, CCR, and PDD datasets in multiple stages (Figure 1), relying on a combination of the following 9 linkage identifiers: date of birth, sex, Social Security number (SSN), zip code of residence, date and type of hematologic malignancy diagnosis, date and type of HCT, and transplantation center. Table 2 provides completeness of each variable by data source, and Table 3 specifies the combinations of identifiers that enabled a match. Most linkage identifiers were very complete (>90%) across all data sources except for SSN (available for 11.2% of CIBMTR patients) and ZIP code of residence (available for 39% of CIBMTR patients) (Table 2).

In the first step, we linked the CCR and PDD admissions data using the CCR’s deterministic and probabilistic algorithms [20] routinely used for research (Figure 1). We then linked all CCR patients with a hematologic malignancy (regardless of linkage status to the PDD) to the CIBMTR. Finally, we linked CIBMTR patients who did not match to the CCR directly to the PDD after restricting the PDD to admissions that had HCT procedure codes. All data elements except hematologic malignancy type and date of diagnosis, which are not available in the PPD, were used in this final linkage.

The National Cancer Institute’s Match*Pro v1.6.1 software [21] was used to conduct the linkages using a probabilistic approach based on the Fellegi and Sunter model [22]. Blocking sensitivity was set to 2, so that records from the data sources were compared if at least 2 blocking variables (SSN, birth date, hematologic malignancy diagnosis date, HCT date, ZIP code of residence, hematologic malignancy type [defined for linkage purposes as leukemia/preleukemia, myeloma, and lymphoma]) were equivalent. Matching variables were used to calculate the m probability (ie, the probability that the 2 fields agree for a matching pair) and u probability (ie, the probability that 2 fields agree for a nonmatching pair) for each field. The match classification filter in Match*Pro was used to customize the classification of matches to our matching criteria in Table 3.

Uncertain matches, many-to-one matches, and nonmatches were manually reviewed, with data for linkage identifiers systematically compared to determine agreement. Manual review was applied to determine agreement between facility names, as names were not standardized within the CIBMTR or across data sources. Facilities were considered to be in agreement if names were in a different order or an acronym was used. In addition, race/ethnicity data from the CIBMTR and CCR were used only for manual review, as all of Match*Pro’s blocking and matching parameters were maxed out with the other parameters.

### Statistical Analyses

We evaluated the relationship of patient-, HCT-, and HCT center-related factors with match status (unmatched or matched to the PDD only versus matched to the CCR with or without the PDD). Data are presented at the patient level of the first HCT by HCT type (allogeneic versus autologous). In addition to descriptive statistics (n, frequency), we constructed polytomous logistic regression models to identify key predictors of match status, first evaluating univariable results and then multivariable results because of correlation among different factors (eg, indication for and age at HCT). Multivariable models included linkage variables, HCT center volume, HCT year, sex, race/ethnicity, age at HCT, and hematologic malignancy type. Results are presented as adjusted odds ratios (ORs) and associated 95% confidence intervals (CIs).

We determined the concordance of broad hematologic malignancy type and subtype between the CIBMTR and CCR. We also determined the concordance of HCT type (allogeneic and autologous) between the CIBMTR and the PDD and CCR. Hematologic malignancy diagnosis and HCT procedure dates were compared using ≤90-day and >90-day thresholds. Finally, we compared the concordance of HCT centers between the CIBMTR and the PDD. Analyses were conducted using SAS version 9.4 (SAS Institute, Cary, NC).

## RESULTS

In a multistep linkage (Figure 1), we attempted to match 22,733 patients in the CIBMTR who underwent 1 or more autologous or allogeneic HCTs (n = 24,938 transplantations) for a hematologic malignancy diagnosed between 1991 and 2016 to data from the CCR and the PDD. A total of 20,228 (89.0%) matched, including 17,707 (77.9%) matched to both datasets, 1179 (5.2%) matched to CCR only, and 1342 (5.9%) matched to PDD only. A total of 2505 (11%) CIBMTR patients remained unmatched.

### Patient and Center Characteristics by Matching Status

**Figure 2.**
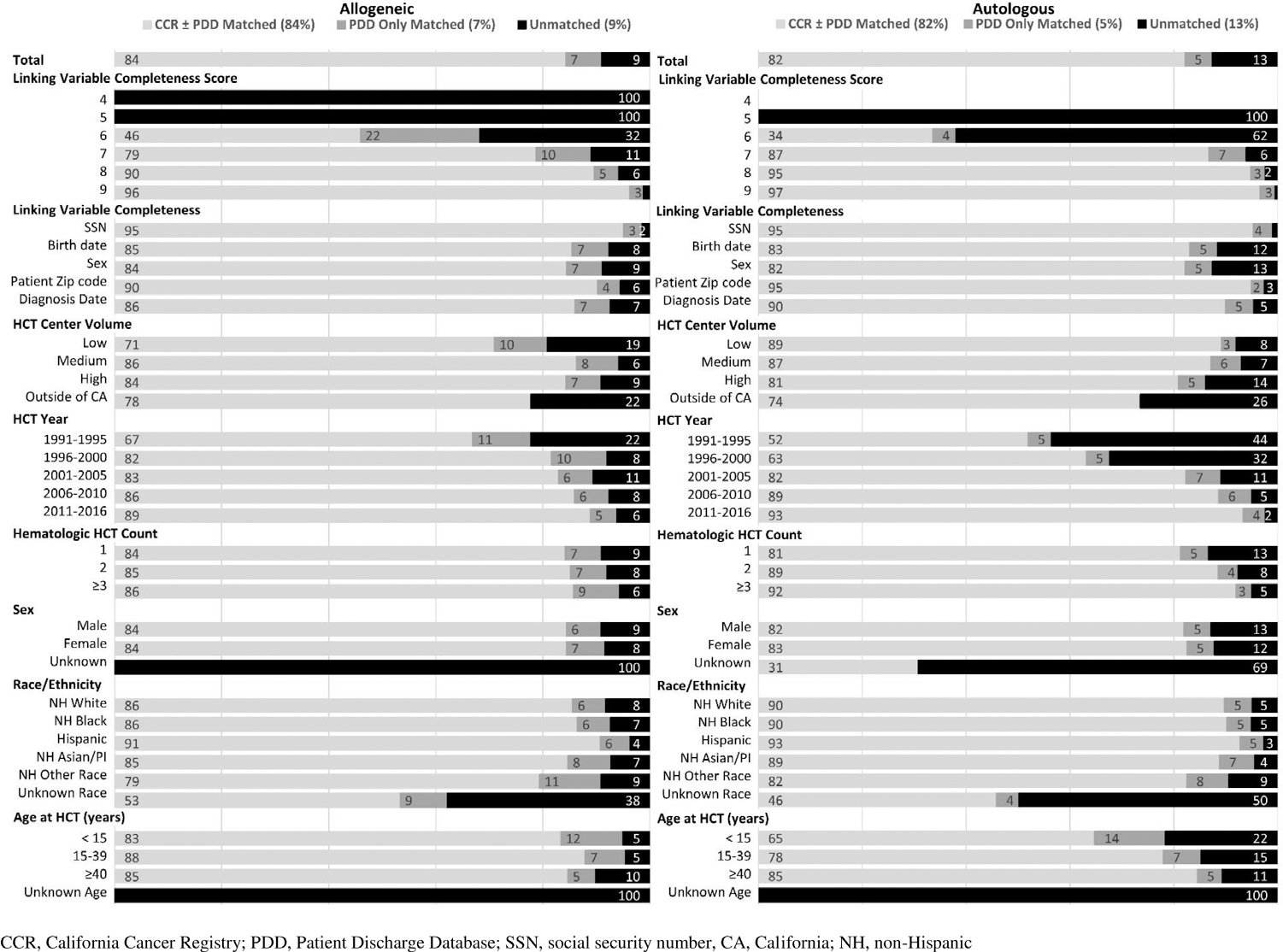
Linkage variable completeness and characteristics of patients (%) who underwent HCT and are registered with the CIBMTR by HCT type and match status. CA, California; NH, non-Hispanic.

Figure 2 presents the availability of linkage variables and characteristics of CIBMTR patients by their matching status. CIBMTR patients who remained unmatched to either CCR or PDD were slightly more likely to have undergone a first autologous HCT (12.6%) rather than a first allogeneic HCT (9.0%). In multivariable models, regardless of HCT type, patients who remained unmatched were more likely to have incomplete data for the linkage variables and to have other/unknown race/ethnicity in the CIBMTR (Table 4). In addition, among patients who underwent allogeneic HCT, those who remained unmatched were most likely to have undergone transplantation between 2001 and 2010 and from a low-volume center or center outside California, to be age ≥40 years at receipt of HCT, and to have undergone transplantation for chronic lymphocytic leukemia/small lymphocytic lymphoma, myelodysplastic syndrome (MDS), or other myeloproliferative neoplasm (MPN). Among autologous HCT recipients, patients who remained unmatched were most likely to have undergone transplantation between 1991 and 2005 and from a medium-volume center or a center outside California, and to have undergone HCT for a plasma cell neoplasm. Results generally were similar for patients who matched to the PDD only, except they also were more likely to have undergone HCT at a younger age (<40 years) (Figure 2, Table 4).

### Concordance of Hematologic Malignancy Type between the CIBMTR and CCR

**Figure 3.**
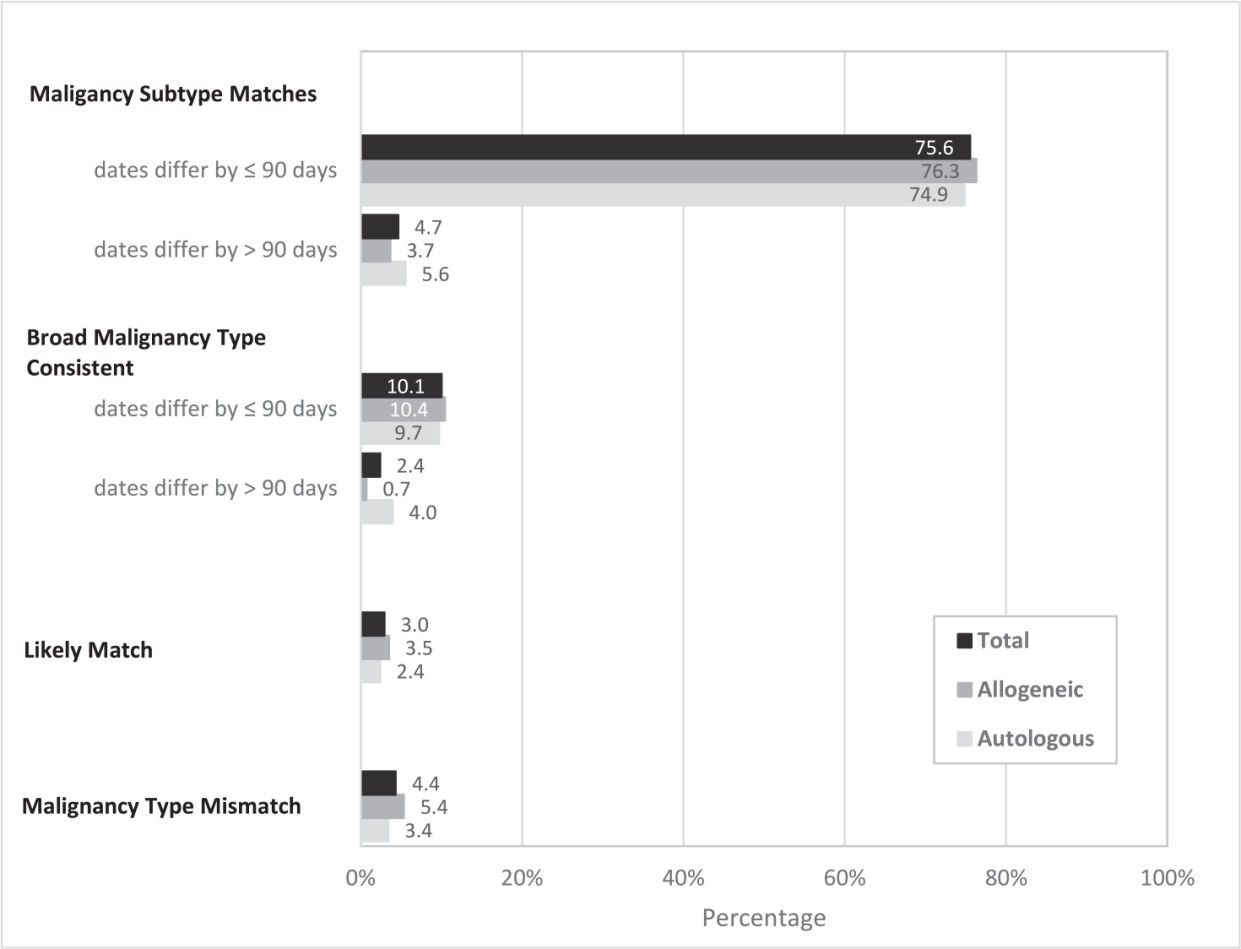
Concordance between CIBMTR indication for HCT and CCR hematologic malignancy type among CIBMTR patients who matched to the CCR. Includes 20,820 CIBMTR-registered HCTs matched to a CCR hematologic cancer. Does not include patients who did not match to the CCR (n = 1447 to the PDD only; n = 2505 unmatched). Broad malignancy type consistent includes one source having a more specific diagnosis and the other having a consistent but less specific diagnosis. Likely match includes known changes in diagnostic criteria, composite diagnoses, and transformations.

We compared the hematologic malignancy diagnoses from the CCR and each HCT indication in the CIBMTR. Because of differences in the specificity of the information and changes in the classification of hematologic malignancies over the last several decades, we considered concordance by broader type (eg, Hodgkin lymphoma) as well as by subtype (eg, nodular sclerosis classical Hodgkin lymphoma) (Supplementary Table S1A, B). The CIBMTR and CCR were concordant for both hematologic malignancy subtype and diagnosis date for 75.6% of HCTs and for broad hematologic malignancy type and date for an additional 10.1% of HCTs, generally because of one source having a more specific diagnosis and the other having a consistent but less specific diagnosis (Figure 3). An additional 7.1% of HCTs were concordant by hematologic malignancy type or subtype, but not by date (diagnosis date >90 days different). For 3.0% of patients, hematologic malignancy types were considered likely matches because they were consistent with known changes in diagnostic criteria (eg, MDS versus acute myelogenous leukemia [AML] occurring within 90 days, owing to a change in the blast count threshold), composite diagnoses (eg, follicular and diffuse large B cell lymphoma occurring within 90 days), or transformations (eg, MDS or MPN to AML, follicular lymphoma or chronic lymphocytic leukemia/small lymphocytic lymphoma to diffuse large B cell lymphoma with diagnosis dates >90 days apart).

### HCT Concordance of CIBMTR HCTs with PDD and CCR Data

**Figure 4.**
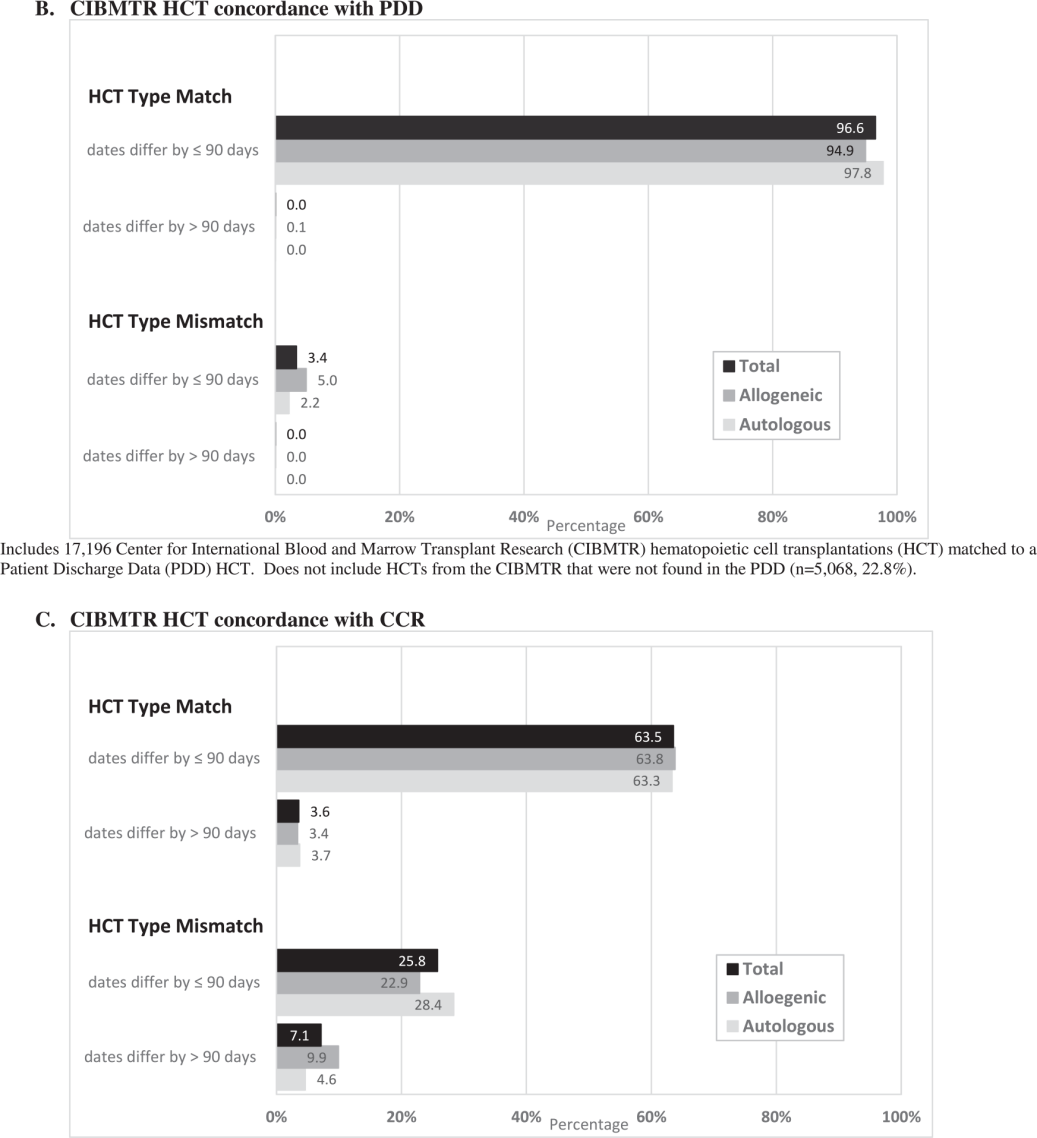
Concordance of HCT type recorded by the CIBMTR with the PDD and CCR among patients who matched with each data source. (A) CIBMTR HCT concordance with the PDD includes 17,196 CIBMTR HCTs matched to a PDD HCT. Does not include HCTs from the CIBMTR that were not found in the PDD (n = 5068; 22.8%). (B) CIBMTR HCT concordance with the CCR. Includes 7214 CIBMTR HCTs matched to a CCR HCT from 2003 diagnoses onward. Does not include HCTs from the CIBMTR that had no HCT recorded as part of their initial treatment in the CCR (n = 7380; 50.6%).

Overall, 77.4% of HCTs, including 69.9% of allogeneic HCTs and 84.3% of autologous HCTs, from the CIBMTR were found in the PDD. Concordance of CIBMTR HCT data with the PDD in terms of both HCT type and date occurred in 96.6% of HCTs (94.9% allogeneic, 97.8% autologous) (Figure 4A). In addition, 98.1% had concordance between reported HCT center in the PDD and the CIBMTR (data not shown). A lower proportion (49.4%) of HCTs from the CIBMTR had HCT recorded as part of their initial treatment in the CCR. For those with an HCT noted in the CCR, concordance in terms of both date and type of HCT was 63.5% (Figure 4B).

## DISCUSSION

In this large data linkage of HCTs performed in California over nearly 3 decades, we successfully matched 89% of patients reported to the CIBMTR to the CCR and/or PDD, which is comparable to or higher than similar linkages, including the linkage of CIBMTR data to Medicare administrative claims data [23] and linkages of state cancer registries with administrative claims data [24,25]. The likelihood of successful matching was significantly reduced if complete identifiers were missing and/or if HCT occurred prior to 2010; hematologic malignancy type and HCT center characteristics also were associated with the likelihood of matching. However, demographic data were generally similar in matched and unmatched patients, suggesting that our linkage was representative of the CIBMTR HCT population in California. The high matching rate in our study allows for the use of these complementary data sources in a range of analyses including investigations into HCT access, healthcare resource utilization, late effects (eg, cardiovascular disease, subsequent malignancies), and cause-specific mortality.

As expected, individuals with fewer reported linkage variables were less likely to match with the CCR. The impact of missing linkage data was observed across individual linkage variables, suggesting that each variable was valuable in the linkage process. A known challenge in performing high-quality linkages is the lack of personal identifiers [26], and our linkage lacked or had limited availability of several key identifiers, including patient name and SSN for CIBMTR patients. In a prior study of 11,358 patients who underwent HCT in 2010 to 2012 in the Medicare claims data, 80% matched to the CIBMTR, even though SSN was missing for 64% [23]. In the present study, although a higher portion of patients with an SSN matched (96%), 71% of patients without an SSN matched with other identifiers, including date of birth, sex, HCT date, and HCT type [23]. In addition, a linkage of the Utah cancer registry with all-payer claims data (82.4% overall linkage) found relatively high matching success without the SSN when other linkage variables were known [25].

Because ZIP code of residence was not available for the majority of CIBMTR patients, it is likely that most of the 11% of patients who did not link to the CCR were not California residents at the time of hematologic malignancy diagnosis. As the CCR only ascertains reportable cancer among those who live in California at the time of diagnosis, out-of-state residents who underwent HCT in California would not match in the CCR. In addition, certain variables used in the linkage were not standardized, such as the name of the reporting transplantation center, resulting in the need for a manual review. Despite these challenges, our process resulted in a high matching rate that approached or surpassed previous large data linkages [24,25]. Improving the completeness of the key identifiers used in this study and including patient names will facilitate high-quality linkages in the future.

We found that the likelihood of successful linkage improved over time, commensurate with the implementation of reporting requirements for allogeneic HCT. In addition to calendar year, the ability to successfully link across data sources varied by hematologic malignancy type and HCT center characteristics. For example, linkage of cases with MDS and MPN was less successful than linkage with AML, which was anticipated given that MDS/MPN were not reportable to the CCR until 2001. For autologous HCT, linkage of multiple myeloma was less successful than other malignancies, even after adjusting for the availability of linkage variables and other patient characteristics. This may be due to performing autologous HCT in the outpatient setting (limiting linkage to the PDD) or may be a consequence of the indolent clinical course in certain patients, allowing for emigration from the state of diagnosis. Finally, transplantation center volume was associated with linkage success in both autologous and allogeneic HCT recipients, with high-volume centers more likely to match, perhaps because of increased familiarity or staffing to conduct CIBMTR data collection and reporting.

Among patients reported to the CIBMTR who matched to the CCR and/or PDD, we observed a very high proportion concordant for hematologic malignancy type and date of diagnosis. In addition, for a small proportion of patients, we found that diagnostic criteria changes, composite diagnoses, and transformations likely impacted the concordance between data sources. We also found that 23% of HCTs from the CIBMTR were not included in the PDD, likely related to the PDD not capturing the HCTs performed in outpatient settings. Moreover, 51% of HCTs from the CIBMTR were not found in the CCR. The CCR findings may be related to HCTs being performed further out from hematologic malignancy diagnosis, given that cancer registries are mandated to capture first-course treatment within approximately 1 year of diagnosis, or underreporting of some cancer treatments, including HCT [15,27–29]. Our findings highlight the importance of augmenting population-based cancer registry data with HCT data from other sources, including PDD, outpatient, or CIBMTR data, to fully capture its utilization.

Although the size and scope of our study are strengths, inconsistencies and inaccuracies in reporting are possible, as in any population-based registry study; however, our high rate of matching and the ability to examine sociodemographic and clinical characteristics across data sources are anticipated to result in improved data quality. The lack of personal identifiers was overcome through a robust approach to the linkage methods and the use of 3 different data sources, which increased the likelihood of matching to at least 1 of the data sources. Finally, the use of cancer registry and hospitalization databases from California might limit the generalizability of our results to other settings that may differ in racial/ethnic and socioeconomic status composition or by referral and transplantation practices. Nonetheless, the linkage methods used in this study can be applied to other geographic regions with cancer registries and statewide hospitalization or all-payer claims databases.

In summary, we have demonstrated the successful linkage between the CIBMTR and 2 state-wide population-based data resources (CCR and PDD) to generate a unique database that can address a host of health outcomes, health services, and clinical research questions. Clinical HCT practices have evolved substantially in recent decades, particularly with the introduction of reduced-intensity conditioning regimens, reduced dependency on radiation-based conditioning, growing use of alternative stem cell donor sources, and expanding approaches for the diagnosis and management of graft-versus-host disease. How these advances have affected access to HCT for patients in need is poorly understood, as few studies contain large populations of both transplantation recipients and non-recipients with hematologic malignancies. In addition, the impact of these changes on long-term adverse effects that may occur many years after HCT (eg, total body irradiation-related subsequent malignancies) is not well understood. Data linkages such as ours that capitalize on the strengths of multiple complementary data sources provide an opportunity to capture information related to cancer diagnosis, treatment, and outcomes that can address these research gaps and serve as a source for emerging questions in cellular therapy.

## Supplementary Material

1

## Figures and Tables

**Table 1:** Hematologic Malignancy Types in the CIBMTR and CCR

CIBMTR	
Disease Codes	Description
10	Acute myelogenous or acute nonlymphocytic leukemia
20	Acute lymphoblastic leukemia
30	Other leukemia
40	Chronic myelogenous leukemia
50	Myelodysplastic/myeloproliferative disorders
80	Other acute leukemia
100	Non-Hodgkin lymphoma
150	Hodgkin lymphoma
170	Plasma cell disorder/multiple myeloma
570	Histiocytic disorders
**CCR**	
**Histology**	Description (Site Recode*)
9650–9667	Hodgkin lymphoma (33011, 33012)
9590–9597, 9670–9671, 9673, 9675, 9678–9680, 9684, 9687–9691, 9695, 9698–9702, 9705, 9708–9709, 9712, 9714–9719, 9724–9729, 9735, 9737–9738, 9811–9818, 9823, 9827, 9837	Non-Hodgkin lymphoma (33041, 33042)
9731–9732, 9734	Myeloma (34000)
9811–9818, 9826, 9835–9837	Acute lymphocytic leukemia (35011)
9823	Chronic lymphocytic leukemia (35012)
9820, 9832–9834, 9940	Other lymphocytic leukemia (35013)
9840, 9861, 9865–9867, 9869, 9871–9874, 9895–9897, 9898, 9910–9911, 9920	Acute myeloid leukemia (35021)
9863, 9875–9876, 9945–9946	Chronic myeloid leukemia (35022)
9860, 9930	Other myeloid/monocytic leukemia (35023)
9891	Acute monocytic leukemia (35031)
9733, 9742, 9800–9801, 9805–9809, 9827, 9931, 9870, 9948, 9963–9964	Other leukemia (35041, 35043)
9761, 9950, 9960–9962, 9971, 9975, 9980, 9982–9987, 9989, 9991–9992	Miscellaneous (37000)

* https://seer.cancer.gov/siterecode/.

**Table 2:** Completeness of Linkage Identifiers in the CIBMTR, CCR, and California PDD

Linkage Identifier	CIBMTR, %	CCR, %	PDD, %*
SSN	11.2	93.7	100.0
ZIP code of residence	39.0	99.5	99.3
Diagnosis date^†^	91.3	90.6	^‡^
Birth date^†^	98.8	99.9	100.0
Sex	99.9	100.0	100.0
Hematologic malignancy type	100.0	100.0	^‡^
HCT type	100.0	91.0^§^	100.0^§^
HCT center	100.0	^‡^	100.0^§^
HCT date^†^	100.0	92.7^§^	100.0^§^

* PDD is restricted to admissions with a valid social security number.

† Completeness is based on full dates (MM/DD/YYYY).

‡ Not utilized.

§ Limited to those with a known transplantation.

**Table 3:** Combinations of Linkage Identifiers to Determine a Match between Patients in the CIBMTR Registry, CCR, and California PDD

Birth Date*	Hematologic Malignancy Diagnosis Date^†^	HCT Date^‡^	Sex^§^	SSN^∥^	HCT Center^¶^	Hematologic Malignancy Type^¶^	ZIP Code**
One of these			✓	✓			
✓	✓	✓				✓	✓
✓	✓	✓			✓	One of these	
✓	✓	100% similarity			One of these		
One of these		✓	✓			✓	✓
✓	✓	Same HCT type, same HCT date not required	✓		✓	One of these	
✓		✓			✓	One of these	
✓		✓	✓		✓		
✓	✓		✓				✓
✓			✓			✓	✓
✓	✓		✓			Both patients have a hematologic cancer	

Each row represents an allowable combination of identifiers

* Birth date was compared using the date comparator with a threshold of .75 and an m probability of .96. Two dates with a calculated similarity greater than or equal to .83 were considered the same date.

† Diagnosis date was compared using a temporal comparator with a threshold of 90 days and an m probability of .96.

‡ HCT date was compared using a temporal comparator with a threshold of 90 days and an m probability of .96. The date of HCT was taken into consideration, even if the type of transplant was different.

§ Sex was compared using the exact comparator with a threshold of 1.00 and an m probability of .95.

∥ SSN was compared using the SSN comparator with a threshold of .76 and an m probability of .95.

¶ Facility name was compared using the overlap comparator with a threshold of .80 and an m probability of .75.#Hematologic malignancy type was compared using the Winkler comparator with a threshold of .80 and an m probability of .95. Type was considered a match if both patients in the pair had leukemia/preleukemia, myeloma, or lymphoma (broad categories).

** ZIP code was compared using the postal code comparator with a threshold of .78 and an m probability of .95

**Table 4:** Multivariable-Adjusted* ORs and Associated 95% CIs of Factors Associated with Match Status (Matched to the PDD Only or Unmatched, Compared with Matched to the CCR) Among Patients Who Underwent HCT in the CIBMTR Registry Stratified by HCT Type

Variables	Allogeneic HCT							Autologous HCT					
	Odds of PDD-Only Matched vs CCR-Matched			Odds of Unmatched vs CCR-Matched			Odds of PDD-Only Matched vs CCR-Matched				Odds of Unmatched vs CCR- Matched		
	OR	95% CI	*P* Value	OR	95% CI	*P* Value		OR	95% CI	*P* Value	OR	95% CI	*P* Value
Linkage variables													
Complete diagnosis date	1.00	Reference		1.00	Reference		1.00		Reference		1.00	Reference	
Missing diagnosis date	3.94	2.34–6.62	<.01	3.58	2.13–6.04	<.01		1.88	1.25–2.83	<.01	21.13	16.13–27.67	<.01
Complete ZIP code	1.00	Reference		1.00	Reference		1.00		Reference		1.00	Reference	
Missing ZIP code	2.79	2.32–3.36	<.01	2.71	2.23–3.30	<.01		3.23	2.50–4.19	<.01	2.12	1.61–2.78	<.01
Complete SSN	1.00	Reference		1.00	Reference		1.00		Reference		1.00	Reference	
Missing SSN	2.13	1.58–2.88	<.01	8.92	5.79–13.73	<.01		1.85	1.29– 2.65	<.01	3.71	1.89–7.30	<.01
HCT center volume													
Low-volume center	.85	.51–1.41	.52	2.09	1.32–3.29	<.01		.46	.23–.92	.03	.68	.42–1.11	.12
Medium-volume center	1.26	1.01–1.57	.04	1.11	.86–1.44	.41		1.08	.88–1.33	.48	1.46	1.17–1.81	<.01
High-volume center	1.00	Reference		1.00	Reference		1.00		Reference		1.00	Reference	
Outside California	.00	–	.95	7.93	5.09–12.35	<.01		.00	–	.96	4.76	2.96–7.66	<.01
HCT year													
1991–1995	1.84	1.35–2.50	.01	1.25	.87–1.78	.23		1.02	.70–1.49	.91	8.47	6.13–11.72	<.01
1996–2000	1.96	1.46–2.64	<.01	1.20	.86–1.66	.29		.96	.70–1.32	.80	4.72	3.48–6.42	<.01
2001–2005	1.09	.82–1.45	.54	1.88	1.45–2.44	<.01		.89	.68–1.16	.37	1.44	1.05–1.97	.02
2006–2010	1.31	1.04–1.65	.02	1.50	1.18–1.90	<.01		.94	.74–1.18	.59	1.03	.75–1.40	.87
2011–2016	1.00	Reference		1.00	Reference		1.00		Reference		1.00	Reference	
Sex													
Male	1.00	Reference		1.00	Reference		1.00		Reference		1.00	Reference	
Female	1.18	1.00–1.40	.05	1.04	.87–1.23	.69		.99	.84–1.16	.87	.94	.81–1.09	.38
Race/ethnicity													
Non-Hispanic white	1.00	Reference		1.00	Reference		1.00		Reference		1.00	Reference	
Non-Hispanic black	1.05	.65–1.68	.86	1.01	.63–1.64	.96		.87	.60–1.26	.45	1.14	.78–1.68	.49
Hispanic	1.06	.82–1.38	.65	.81	.60–1.09	.17		.97	.72–1.31	.83	1.25	.85–1.85	.26
Non-Hispanic Asian/Pacific Islander	1.25	.96–1.64	.10	1.00	.74–1.35	1.00		1.38	1.03–1.84	.03	1.29	.90–1.87	.17
Non-Hispanic other	1.25	.93–1.69	.14	1.63	1.16–2.31	<.01		1.25	.86–1.83	.25	1.33	.89–1.96	.16
Unknown	.85	.56–1.30	.46	1.92	1.29–2.86	<.01		.89	.62–1.27	.50	1.40	1.08–1.81	.01
Age at HCT													
<15 yr	2.29	1.79–2.91	<.01	.74	.54–1.00	.05		3.29	2.02–5.37	<.01	1.65	.98–2.79	.06
15–39 yr	1.53	1.24–1.88	<.01	.70	.56–.89	<.01		1.51	1.19–1.91	<.01	1.11	.89–1.38	.36
≥40 yr	1.00	Reference		1.00	Reference		1.00		Reference		1.00	Reference	
Hematologic malignancy type													
Hodgkin lymphoma	NA			NA			.93		.70–1.23	.59	.23	.17–.31	<.01
Diffuse large B cell lymphoma	1.08	.57–2.05	.81	.28	.09–.89	.03		.75	.59–.96	.02	.32	.24–.42	<.01
Mantle cell lymphoma	NA			NA			.84		.57–1.23	.37	.29	.17–.52	<.01
Follicular lymphoma	NA			NA			.68		.40–1.17	.16	.35	.21–.58	<.01
T cell lymphoma	NA			NA			.69		.42–1.14	.15	.31	.16–.60	<.01
Plasma cell neoplasm	NA			NA			1.00		Reference		1.00	Reference	
AML	1.00	Reference		1.00	Reference			.63	.44–.90	.01	.13	.09–.18	<.01
CLL/SLL	1.18	.56–2.46	.67	2.76	1.72–4.43	<.01		NA			NA		
Chronic myelogenous leukemia	1.15	.84–1.57	.39	1.04	.73–1.47	.84		NA			NA		
Other leukemia	1.44	.81–2.55	.21	.85	.34–2.12	.72		.99	.29–3.41	.98	.28	.12–.68	<.01
Other lymphoma/lymphoid leukemia	.90	.62–1.32	.60	1.11	.78–1.56	.57		.68	.49–.95	.02	.27	.21–.34	<.01
Precursor leukemia/lymphoma	1.01	.80–1.28	.91	.46	.32–.66	<.01		NA			NA		
MDS	8.88	6.88–11.46	<.01	20.11	15.80–25.60	<.01		NA			NA		
Other MPNs	7.25	5.13–10.25	<.01	18.33	13.60–24.69	<.01		NA			NA		

CLL/SLL indicates chronic lymphocytic leukemia/small lymphocytic lymphoma. MPN indicates myeloproliferative neoplasms.

* Adjusted for all variables in the table; patients with unknown sex (n = 32) or unknown age (n = 292) were excluded from the models.
