# Supplementary material for: Linking the Center for International Blood and Marrow Transplant Research Registry to the California Cancer Registry and California Hospital Patient Discharge Data
Source: Transplant Cell Ther. Author manuscript; Available in PMC 2023 Jul 18. (PMC10353452; doi:10.1016/j.jtct.2022.09.016)
Supplement: 1 [file NIHMS1906908-supplement-1.docx]

| **Supplementary Table S1A. Center for International Blood and Marrow Transplant Research (CIBMTR) disease and sub-disease classifications to create broad and detailed categories for hematopoietic cell transplant indication.** | | | | |
| --- | --- | --- | --- | --- |
| **Broad Category** | **Detailed Category** | **CIBMTR Disease** | **CIBMTR Sub-disease** |  |
| **Hodgkin Lymphoma** | Hodgkin Lymphoma | Hodgkin lymphoma | HD, NOS |  |
|  | Hodgkin Lymphoma | Hodgkin lymphoma | HD other, specify |  |
|  | Lymphocyte-rich | Hodgkin lymphoma | HD lymphocyte-rich |  |
|  | Mixed cellularity | Hodgkin lymphoma | HD mixed cellularity |  |
|  | Lymphocyte-depleted | Hodgkin lymphoma | HD lymphocyte depleted |  |
|  | Nodular sclerosis | Hodgkin lymphoma | HD nodular sclerosis |  |
|  | Nodular lymphocyte predominant Hodgkin lymphoma | Hodgkin lymphoma | nodular lymphocyte predominant Hodgkin lymphoma |  |
| **Precursor leukemia/lymphoma** | Precursor leukemia/lymphoma, B-cell | Acute lymphoblastic leukemia | ALL Mature B-cell (L3) |  |
|  | Precursor leukemia/lymphoma, B-cell | Acute lymphoblastic leukemia | ALL, B-lineage, NOS |  |
|  | Precursor leukemia/lymphoma, B-cell | Acute lymphoblastic leukemia | B-lymphoblastic leukemia/lymphoma, BCR-ABL1-like |  |
|  | Precursor leukemia/lymphoma, B-cell | Acute lymphoblastic leukemia | B-cell ALL,NOS |  |
|  | Precursor leukemia/lymphoma, B-cell | Acute lymphoblastic leukemia | t(9;22)(q34.1;q11.2); BCR/ABL1 |  |
|  | Precursor leukemia/lymphoma, B-cell | Acute lymphoblastic leukemia | t(v;11q23.3); KMT2A rearranged |  |
|  | Precursor leukemia/lymphoma, B-cell | Acute lymphoblastic leukemia | t(1;19)(q23;p13.3);TCF3-PBX1 |  |
|  | Precursor leukemia/lymphoma, B-cell | Acute lymphoblastic leukemia | t(12;21)(p13.2;q22.1); ETV6-RUNX1 |  |
|  | Precursor leukemia/lymphoma, B-cell | Non-Hodgkin lymphoma | Precursor B-lymphoblastic |  |
|  | Precursor leukemia/lymphoma, B-cell | Non-Hodgkin lymphoma | B-cell ALL,NOS |  |
|  | Precursor leukemia/lymphoma, T/NK-cell | Acute lymphoblastic leukemia | Natural killer (NK)- cell lymphoblastic leukemia/lymphoma |  |
|  | Precursor leukemia/lymphoma, T/NK-cell | Acute lymphoblastic leukemia | ALL T-lineage/precursor T-cell ALL |  |
|  | Precursor leukemia/lymphoma, T/NK-cell | Non-Hodgkin lymphoma | Precursor T-lymphoblastic |  |
|  | Precursor leukemia/lymphoma, unknown lineage | Acute lymphoblastic leukemia | ALL other,specify |  |
|  | Precursor leukemia/lymphoma, unknown lineage | Acute lymphoblastic leukemia | Hyperdiploidy (51-65 chromosomes) |  |
|  | Precursor leukemia/lymphoma, unknown lineage | Acute lymphoblastic leukemia | Hypodiploidy (<45 chromosomes) |  |
|  | Precursor leukemia/lymphoma, unknown lineage | Acute lymphoblastic leukemia | ALL, not otherwise specified |  |
| **Chronic/Small lymphocytic leukemia/lymphoma** | Chronic/Small lymphocytic leukemia/lymphoma | Other leukemia | CLL Chronic lymphocytic leukemia, NOS |  |
|  | Chronic/Small lymphocytic leukemia/lymphoma | Other leukemia | CLL B-cell/lym small lymphocytic |  |
|  | Chronic/Small lymphocytic leukemia/lymphoma | Non-Hodgkin lymphoma | CLL Chronic lymphocytic leukemia, NOS |  |
|  | Chronic/Small lymphocytic leukemia/lymphoma | Non-Hodgkin lymphoma | NHL small lymphocytic |  |
| **Prolymphocytic leukemia** | Prolymphocytic leukemia | Other leukemia | PLL Prolymphocytic leukemia |  |
|  | Prolymphocytic leukemia | Other leukemia | PLL B-cell |  |
|  | Prolymphocytic leukemia | Other leukemia | PLL T-cell |  |
| **Mantle-cell lymphoma** | 2(a)2.1.3. Mantle-cell lymphoma | Non-Hodgkin lymphoma | NHL mantle cell |  |
| **Lymphoplasmacytic lymphoma/Waldenstrom** | 2(a)2.2. Lymphoplasmacytic lymphoma/Waldenstrom | Non-Hodgkin lymphoma | NHL small lymphoplasmacytic |  |
|  | 2(a)2.2. Lymphoplasmacytic lymphoma/Waldenstrom | Non-Hodgkin lymphoma | Waldenstrom macroglobulinemia |  |
| **Diffuse large B-cell lymphoma** | Diffuse large B-cell lymphoma | Non-Hodgkin lymphoma | NHL diffuse,smalll cleaved cell |  |
|  | Diffuse large B-cell lymphoma | Non-Hodgkin lymphoma | NHL diffuse,mixed,small and large cell |  |
|  | Diffuse large B-cell lymphoma | Non-Hodgkin lymphoma | NHL diffuse, large B-cell |  |
|  | Diffuse large B-cell lymphoma | Non-Hodgkin lymphoma | NHL large cell,immunoblastic |  |
|  | Diffuse large B-cell lymphoma | Non-Hodgkin lymphoma | NHL histiocytic |  |
|  | Diffuse large B-cell lymphoma | Non-Hodgkin lymphoma | T-cell / histiocytic rich large B-cell lymphoma |  |
|  | Diffuse large B-cell lymphoma | Non-Hodgkin lymphoma | Intravascular large B-cell lymphoma |  |
|  | Diffuse large B-cell lymphoma | Non-Hodgkin lymphoma | Primary effusion lymphoma |  |
|  | Diffuse large B-cell lymphoma | Non-Hodgkin lymphoma | Primary mediastinal large B-cell |  |
|  | Diffuse large B-cell lymphoma/Burkitt | Non-Hodgkin lymphoma | B-cell unclass. between DLBCL and Burkitt |  |
|  | Diffuse large B-cell lymphoma/Hodgkin lymphoma | Non-Hodgkin lymphoma | B-cell unclass. between DLBCL and hodgkin |  |
| **Burkitt lymphoma/leukemia** | Burkitt lymphoma/leukemia | Non-Hodgkin lymphoma | Burkitt lym/Burkitt cell leukemia |  |
| **Marginal-zone lymphoma** | Splenic marginal-zone lymphoma | Non-Hodgkin lymphoma | Splenic marginal zone B-cell |  |
|  | Extranodal marginal-zone lymphoma, MALT type | Non-Hodgkin lymphoma | Extranodal marginal zone B-cell of MALT |  |
|  | Nodal marginal-zone lymphoma | Non-Hodgkin lymphoma | Nodal marginal zone B-cell |  |
| **Follicular lymphoma** | Follicular lymphoma | Non-Hodgkin lymphoma | NHL follicular,predominantly small cleaved cell |  |
|  | Follicular lymphoma | Non-Hodgkin lymphoma | NHL follicular,mixed,small cleaved and large cell |  |
|  | Follicular lymphoma | Non-Hodgkin lymphoma | NHL follicular,predominantly large cell |  |
|  | Follicular lymphoma | Non-Hodgkin lymphoma | Follicular, predominantly large cell Grade IIIA |  |
|  | Follicular lymphoma | Non-Hodgkin lymphoma | Follicular, predominantly large cell Grade IIIB |  |
|  | Follicular lymphoma | Non-Hodgkin lymphoma | Follicular unknown grade |  |
| **Hairy-cell leukemia** | Hairy-cell leukemia | Other leukemia | Hairy cell leukemia |  |
| **Plasma cell neoplasms** | Plasma cell neoplasms | Plasma cell disorder/Multiple Myeloma | Plasma cell disorder, NOS |  |
|  | Plasma cell neoplasms | Plasma cell disorder/Multiple Myeloma | Multiple myeloma, NOS |  |
|  | Plasma cell neoplasms | Plasma cell disorder/Multiple Myeloma | Plasma cell leukemia |  |
|  | Plasma cell neoplasms | Plasma cell disorder/Multiple Myeloma | Amyloidosis |  |
|  | Plasma cell neoplasms | Plasma cell disorder/Multiple Myeloma | Solitary plasmacytoma |  |
|  | Plasma cell neoplasms | Plasma cell disorder/Multiple Myeloma | Osteosclerotic myeloma/POEMS syndrome |  |
|  | Plasma cell neoplasms | Plasma cell disorder/Multiple Myeloma | Light chain deposition disease |  |
|  | Plasma cell neoplasms | Plasma cell disorder/Multiple Myeloma | Other plasma cell disorder, specify |  |
|  | Plasma cell neoplasms | Plasma cell disorder/Multiple Myeloma | TED Multiple myeloma-IgG |  |
|  | Plasma cell neoplasms | Plasma cell disorder/Multiple Myeloma | TED Multiple myeloma-IgA |  |
|  | Plasma cell neoplasms | Plasma cell disorder/Multiple Myeloma | TED Multiple myeloma-IgD |  |
|  | Plasma cell neoplasms | Plasma cell disorder/Multiple Myeloma | TED Multiple myeloma-IgE |  |
|  | Plasma cell neoplasms | Plasma cell disorder/Multiple Myeloma | Multiple myeloma-IgM |  |
|  | Plasma cell neoplasms | Plasma cell disorder/Multiple Myeloma | TED Mult myeloma-light chain |  |
|  | Plasma cell neoplasms | Plasma cell disorder/Multiple Myeloma | TED Mult myeloma-non-secretory |  |
|  | Plasma cell neoplasms | Plasma cell disorder/Multiple Myeloma | MYE secretory type unk |  |
| **Mycosis fungoides/Sezary syndrome** | Mycosis fungoides/Sezary syndrome | Non-Hodgkin lymphoma | NHL mycosis fungoides/Sezary syndrome |  |
|  | Mycosis fungoides/Sezary syndrome | Non-Hodgkin lymphoma | Mycosis fungoides |  |
|  | Mycosis fungoides/Sezary syndrome | Non-Hodgkin lymphoma | Sezary syndrome |  |
| **Peripheral T-cell lymphoma** | Peripheral T-cell lymphoma | Non-Hodgkin lymphoma | Peripheral T-cell lymphoma, NOS |  |
|  | Angioimmunoblastic T-cell lymphoma | Non-Hodgkin lymphoma | Angioimmunoblastic T-cell lymphoma |  |
|  | Subcutaneous panniculitis-like T-cell lymph | Non-Hodgkin lymphoma | Subcutaneous panniculitis T-cell |  |
|  | Anaplastic large cell lymphoma, T-/Null-cell | Non-Hodgkin lymphoma | NHL large cell anaplastic, Ki 1+ |  |
|  | Anaplastic large cell lymphoma, T-/Null-cell | Non-Hodgkin lymphoma | Anaplastic large-cell lymphoma (ALCL), ALK positive |  |
|  | Anaplastic large cell lymphoma, T-/Null-cell | Non-Hodgkin lymphoma | Anaplastic large-cell lymphoma (ALCL), ALK negative |  |
|  | Anaplastic large cell lymphoma, T-/Null-cell | Non-Hodgkin lymphoma | Anaplas LC,T/N cell, systemic |  |
|  | Hepatosplenic T-cell lymphoma | Non-Hodgkin lymphoma | Hepatosplenic gamma-delta T-cell |  |
|  | Enteropathy-type T-cell lymphoma | Non-Hodgkin lymphoma | Enteropathy-type T-cell lymphoma |  |
| **Primary cutaneous anaplastic large cell lymphoma** | Primary cutaneous anaplastic large cell lymphoma | Non-Hodgkin lymphoma | Anaplas LC,T/N cell, cutaneous |  |
| **Adult T-cell leukemia/lymphoma** | Adult T-cell leukemia/lymphoma | Non-Hodgkin lymphoma | Adult T-cell lymphoma/leukemia |  |
| **NK/T-cell lymphoma, nasal-type/aggressive NK leukemia** | NK/T-cell lymphoma, nasal-type/aggresive NK leukemia | Non-Hodgkin lymphoma | ALL Aggressive NK-cell Lk |  |
|  | NK/T-cell lymphoma, nasal-type/aggresive NK leukemia | Non-Hodgkin lymphoma | Extranodal NK-T-cell |  |
| **T-cell large granular lymphocytic leukemia** | T-cell large granular lymphocytic leukemia | Non-Hodgkin lymphoma | Large granular lymphocytic leukemia |  |
| **Composite** | Composite NHL | Non-Hodgkin lymphoma | NHL composite,specify |  |
| **Non-Hodgkin lymphoma (NHL), not otherwise specified (NOS)** | NHL, NOS | Non-Hodgkin lymphoma | NHL Low Grade |  |
|  | NHL, NOS | Non-Hodgkin lymphoma | NHL Intermediate Grade |  |
|  | NHL, NOS | Non-Hodgkin lymphoma | NHL High Grade |  |
|  | NHL, NOS | Non-Hodgkin lymphoma | NHL, NOS |  |
|  | NHL, NOS | Non-Hodgkin lymphoma | NHL snc, unclassified |  |
|  | NHL, NOS | Non-Hodgkin lymphoma | NHL snc, non-burkitt |  |
|  | NHL, NOS | Non-Hodgkin lymphoma | NHL other, specify |  |
|  | NHL, NOS | Non-Hodgkin lymphoma | Tranformed low grade lymphoma |  |
|  | NHL, NOS | Non-Hodgkin lymphoma | Other B-cell, spec |  |
|  | NHL, NOS | Non-Hodgkin lymphoma | High-grade B-cell lymphoma |  |
|  | NHL, NOS | Non-Hodgkin lymphoma | Other T/NK-cell lymphoma, specify |  |
|  | Central nervous system lymphoma | Non-Hodgkin lymphoma | Primary CNS lymphoma |  |
| **Lymphoma, NOS** | Lymphoma, NOS | Other leukemia | CLL T-cell |  |
| **Acute myelogenous leukemia (AML)** | Acute myelogenous leukemia | Acute myelogenous leukemia or ANLL | AML with mutated NPM1 |  |
|  | Acute myelogenous leukemia | Acute myelogenous leukemia or ANLL | AML with t(9;11) (p22.3;q23.3);MLLT 3-KMT2A |  |
|  | Acute myelogenous leukemia | Acute myelogenous leukemia or ANLL | AML with t(6;9) (p23;q34.1); DEK-NUP214 |  |
|  | Acute myelogenous leukemia | Acute myelogenous leukemia or ANLL | AML with inv(3) (q21.3;q26.2) or t(3;3) (q21.3;q26.2);GATA2,MECOM |  |
|  | Acute myelogenous leukemia | Acute myelogenous leukemia or ANLL | AML (megakaryoblastic) with t(1;22) (p13.3;q13.3);RBM15-MKL1 |  |
|  | Acute myelogenous leukemia | Acute myelogenous leukemia or ANLL | Therapy related AML (t-AML) |  |
|  | Acute myelogenous leukemia | Acute myelogenous leukemia or ANLL | AML or ANLL, NOS |  |
|  | Acute myelogenous leukemia | Acute myelogenous leukemia or ANLL | M2 Myelocytic |  |
|  | Acute myelogenous leukemia | Acute myelogenous leukemia or ANLL | M4 Myelomonocytic |  |
|  | Acute myelogenous leukemia | Acute myelogenous leukemia or ANLL | Granulocytic sarcoma |  |
|  | Acute myelogenous leukemia | Acute myelogenous leukemia or ANLL | AML other,specify |  |
|  | Acute myelogenous leukemia | Acute myelogenous leukemia or ANLL | AML Transformed from MDS |  |
|  | Acute myelogenous leukemia | Acute myelogenous leukemia or ANLL | AML, not otherwise specified |  |
|  | Acute myelogenous leukemia | Acute myelogenous leukemia or ANLL | AML with t(8;21)(q22;q22.1)RUNX1-RUNX1T1 |  |
|  | Acute myelogenous leukemia | Acute myelogenous leukemia or ANLL | AML with inv(16)(p13.1;1q22)or t(16;16)(p13.1;q22);CBFB-MYH11 |  |
|  | Acute myelogenous leukemia | Acute myelogenous leukemia or ANLL | AML with 11q23 (MLL) abnormalities |  |
|  | Acute myelogenous leukemia | Acute myelogenous leukemia or ANLL | AML with myelodysplasia-related changes |  |
|  | Acute myelogenous leukemia | Acute myelogenous leukemia or ANLL | AML minimally differentiated (M0) |  |
|  | Acute myelogenous leukemia | Acute myelogenous leukemia or ANLL | AML without maturation (M1) |  |
|  | Acute myelogenous leukemia | Acute myelogenous leukemia or ANLL | AML with maturation (M2) |  |
|  | Acute myelogenous leukemia | Acute myelogenous leukemia or ANLL | acute myelomonocytic leukemia (M4) |  |
|  | Acute myelogenous leukemia | Acute myelogenous leukemia or ANLL | acute monoblastic/monocytic leukemia (M5) |  |
|  | Acute myelogenous leukemia | Acute myelogenous leukemia or ANLL | acute erythroid leukemia (M6) |  |
|  | Acute myelogenous leukemia | Acute myelogenous leukemia or ANLL | acute megakaryoblastic leukemia (M7) |  |
|  | Acute myelogenous leukemia | Acute myelogenous leukemia or ANLL | acute panmyelosis with myelofibrosis |  |
|  | Acute myelogenous leukemia | Acute myelogenous leukemia or ANLL | myeloid sarcoma |  |
|  | Acute myelogenous leukemia | Myelodysplastic/myeloprolifterative disorders (please classi | AML, not otherwise specified |  |
|  | Acute promyelocytic leukemia | Acute myelogenous leukemia or ANLL | APL with PML-RARA |  |
|  | Refractory anemia excess blasts in transformation | Myelodysplastic/myeloprolifterative disorders (please classi | RAEBT Refractory anemia excess blasts in transformation |  |
| **Chronic myelogenous leukemia (CML)** | Chronic myelogenous leukemia | Chronic myelogenous leukemia | CML Ph unknown, BCR/ABL unknown |  |
|  | Chronic myelogenous leukemia | Chronic myelogenous leukemia | Ph+, BCR/ABL+ |  |
|  | Chronic myelogenous leukemia | Chronic myelogenous leukemia | Ph+, BCR/ABL- |  |
|  | Chronic myelogenous leukemia | Chronic myelogenous leukemia | Ph+, BCR/ABL unknown |  |
|  | Chronic myelogenous leukemia | Chronic myelogenous leukemia | Ph-, BCR/ABL+ |  |
|  | Chronic myelogenous leukemia | Chronic myelogenous leukemia | Ph unknown, BCR/ABL+ |  |
| **Leukemia, NOS** | Leukemia, NOS | Other leukemia | Other leukemia, NOS |  |
|  | Leukemia, NOS | Other leukemia | Other leukemia, specify |  |
|  | Leukemia, NOS | Other acute leukemia | Acute undifferentiated leukemia |  |
|  | Leukemia, NOS | Other acute leukemia | Other Acute leukemia,spec. |  |
| **Myelodysplastic syndrome (MDS)** | Myelodysplastic syndrome | Myelodysplastic/myeloprolifterative disorders | MDS, NOS |  |
|  | Myelodysplastic syndrome | Myelodysplastic/myeloprolifterative disorders | RA Refractory anemia |  |
|  | Myelodysplastic syndrome | Myelodysplastic/myeloprolifterative disorders | RAEB Refractory anemia excess blasts |  |
|  | Myelodysplastic syndrome | Myelodysplastic/myeloprolifterative disorders | RARS Acquired idiopathic sideroblastic anemia |  |
|  | Myelodysplastic syndrome | Myelodysplastic/myeloprolifterative disorders | RAEB-1 |  |
|  | Myelodysplastic syndrome | Myelodysplastic/myeloprolifterative disorders | RAEB-2 |  |
|  | Myelodysplastic syndrome | Myelodysplastic/myeloprolifterative disorders | RCMD |  |
|  | Myelodysplastic syndrome | Myelodysplastic/myeloprolifterative disorders | RCMD / RS |  |
|  | Myelodysplastic syndrome | Myelodysplastic/myeloprolifterative disorders | 5q-syndrome |  |
|  | Myelodysplastic syndrome | Myelodysplastic/myeloprolifterative disorders | Other MDS, spec |  |
| **Myelodysplastic/myeloprolifterative disorders (MDS/MPN)** | Myelodysplastic/myeloprolifterative disorders | Myelodysplastic/myeloprolifterative disorders | Myelodysplastic/myeloproliferative neoplasm,unclassifiable |  |
|  | Myelodysplastic/myeloprolifterative disorders | Myelodysplastic/myeloprolifterative disorders | Other myelodysplasia or myeloproliferative disorder |  |
|  | Juvenile myelomonocytic leukemia | Myelodysplastic/myeloprolifterative disorders (please classi | Juvenile CML |  |
|  | Atypical chronic myelogenous leukemia | Myelodysplastic/myeloprolifterative disorders (please classi | Ph-, BCR/ABL-,Atypical CML NOS |  |
|  | Atypical chronic myelogenous leukemia | Myelodysplastic/myeloprolifterative disorders (please classi | Ph-, BCR/ABL unknown,Atypical CML NOS |  |
|  | Atypical chronic myelogenous leukemia | Myelodysplastic/myeloprolifterative disorders | Ph unknown BCR/ABL unknown, Atypical CML NOS |  |
|  | Chronic myelomonocytic leukemia | Myelodysplastic/myeloprolifterative disorders | CMMoL Chronic myelomonocytic leukemia |  |
| **Other myeloproliferative neoplasms** | Other myeloproliferative neoplasms | Myelodysplastic/myeloprolifterative disorders | Myeloproliferative neoplasm (MPN), unclassifiable, MPS, NOS |  |
|  | Polycythemia vera | Myelodysplastic/myeloprolifterative disorders | Polycythemia vera |  |
|  | Primary myelofibrosis | Myelodysplastic/myeloprolifterative disorders | Myelofibrosis with myeloid metaplasia |  |
|  | Primary myelofibrosis | Myelodysplastic/myeloprolifterative disorders | Acute Myelofib/Myelosclerosis |  |
|  | Primary myelofibrosis | Myelodysplastic/myeloprolifterative disorders | Other myelofibrosis or myelosclerosis |  |
|  | Primary myelofibrosis | Myelodysplastic/myeloprolifterative disorders | chronic idiopathic myelofibrosis |  |
| **Biphenotypic, bilineage or hybrid leukemia** | Biphenotypic, bilineage or hybrid leukemia | Other acute leukemia | Biphenotypic, bilineage or hybrid leukemia |  |
| **Blastic plasmacytoid dendritic cell neoplasm** | Blastic plasmacytoid dendritic cell neoplasm | Acute myelogenous leukemia or ANLL | Blastic Plasmacytoid dendritic cell neoplasm |  |
|  | Blastic plasmacytoid dendritic cell neoplasm | Other acute leukemia | Blastic Plasmacytoid dendritic cell neoplasm |  |
| **Histiocytic disorders** | Langerhans Cell Histiocytosis | Histiocytic disorders | Langerhans Cell Histiocytosis |  |

| **Supplementary Table S1B. California Cancer Registry (CCR) histology classifications to create broad and detailed categories for cancer diagnosis.** | | |
| --- | --- | --- |
| **Broad Category** | **Broad and Detailed Category** | **CCR Histology** |
| **Hodgkin Lymphoma** | Hodgkin Lymphoma | 9650: Hodgkin lymphoma, NOS |
|  | Lymphocyte-rich | 9651: Hodgkin lymphoma, lymphocyte-rich |
|  | Mixed cellularity | 9652: Hodgkin lymphoma, mixed cellularity, NOS |
|  | Lymphocyte-depleted | 9653: Hodgkin lymphoma, lymphocytic deplet., NOS |
|  | Lymphocyte-depleted | 9655: Hodgkin lymphoma, lymphocyt. deplet., reticular |
|  | Nodular sclerosis | 9663: Hodgkin lymphoma, nodular sclerosis, NOS |
|  | Nodular sclerosis | 9664: Hodgkin lymphoma, nod. scler., cellular phase |
|  | Nodular sclerosis | 9665: Hodgkin lymphoma, nod. scler., grade 1 |
|  | Nodular sclerosis | 9667: Hodgkin lymphoma, nod. scler., grade 2 |
|  | Nodular lymphocyte predominant Hodgkin lymphoma | 9659: Hodgkin lymph., nodular lymphocyte predom. |
| **Precursor leukemia/lymphoma** | Precursor leukemia/lymphoma, B-cell | 9728: Precursor B-cell lymphoblastic lymphoma |
|  | Precursor leukemia/lymphoma, B-cell | 9811: B lymphoblastic leukemia/lymphoma, NOS |
|  | Precursor leukemia/lymphoma, B-cell | 9812: Leukemia/lymphoma with t(9;22)(q34;q11.2);BCR-ABL1 |
|  | Precursor leukemia/lymphoma, B-cell | 9813: Leukemia/lymphoma with t(v;11q23);MLL rearranged |
|  | Precursor leukemia/lymphoma, B-cell | 9814: Leukemia/lymphoma with t(12;21)(p13;q22);TEL-AML1(ETV6 |
|  | Precursor leukemia/lymphoma, B-cell | 9815: B lymphoblastic leukemia/lymphoma with hyperdiploidy |
|  | Precursor leukemia/lymphoma, B-cell | 9817: B lymphblastic leukemia/lymphoma with t(5;14)(q31;q32) |
|  | Precursor leukemia/lymphoma, B-cell | 9818: Leukemia/lymphoma with t(1;19)(q23;p13.3); E2A PBX1 (T |
|  | Precursor leukemia/lymphoma, B-cell | 9836: Precursor B-cell lymphoblastic leukemia |
|  | Precursor leukemia/lymphoma, T/NK-cell | 9729: Precursor T-cell lymphoblastic lymphoma |
|  | Precursor leukemia/lymphoma, T/NK-cell | 9837: T lymphoblastic leukemia/lymphoma |
|  | Precursor leukemia/lymphoma, unknown lineage | 9727: Precursor cell lymphoblastic lymphoma, NOS |
|  | Precursor leukemia/lymphoma, unknown lineage | 9816: Leukemia/lymphoma with hypodiploidy (hypodiploid ALL) |
|  | Precursor leukemia/lymphoma, unknown lineage | 9835: Precursor cell lymphoblastic leukemia, NOS |
| **Chronic/Small lymphocytic leukemia/lymphoma** | Chronic/Small lymphocytic leuk/lymph | 9670: ML, small B lymphocytic, NOS |
|  | Chronic/Small lymphocytic leuk/lymph | 9823: Chronic lymphocytic leukemia/small lymphocytic lymphom |
| **Prolymphocytic leukemia** | Prolymphocytic leukemia | 9832: Prolymphocytic leukemia, NOS |
|  | Prolymphocytic leukemia | 9833: Prolymphocytic leukemia, B-cell type |
|  | Prolymphocytic leukemia | 9834: Prolymphocytic leukemia, T-cell type |
| **Mantle-cell lymphoma** | Mantle-cell lymphoma | 9673: Mantle cell lymphoma |
| **Lymphoplasmacytic lymphoma/Waldenstrom** | Lymphoplasmacytic lymphoma/Waldenstrom | 9671: ML, lymphoplasmacytic |
|  | Lymphoplasmacytic lymphoma/Waldenstrom | 9761: Waldenstrom macroglobulinemia |
| **Diffuse large B-cell lymphoma (DLBCL)** | Diffuse large B-cell lymphoma (DLBCL) | 9680: ML, large B-cell, diffuse |
|  | Diffuse large B-cell lymphoma (DLBCL) | 9684: ML, large B-cell, diffuse, immunoblastic, NOS |
|  | Diffuse large B-cell lymphoma (DLBCL) | 9688: T-cell histiocyte rich large B-cell lymphoma |
|  | Diffuse large B-cell lymphoma (DLBCL) | 9712: Intravascular large B-cell lymphoma |
|  | Diffuse large B-cell lymphoma (DLBCL) | 9735: Plasmablastic lymphoma |
|  | Diffuse large B-cell lymphoma (DLBCL) | 9737: ALK positive large B-cell lymphoma |
|  | Primary effusion lymphoma | 9678: Primary effusion lymphoma |
|  | Mediastinal large B-cell lymphoma | 9679: Mediastinal large B-cell lymphoma |
| **Burkitt lymphoma/leukemia** | Burkitt lymphoma/leukemia | 9687: Burkitt lymphoma, NOS |
|  | Burkitt lymphoma/leukemia | 9826: Burkitt cell leukemia |
| **Marginal-zone lymphoma (MZL)** | Splenic MZL | 9689: Splenic marginal zone B-cell lymphoma |
|  | Splenic MZL | 9699: Marginal zone B-cell lymphoma, NOS |
|  | Extranodal MZL, MALT type | 9699: Marginal zone B-cell lymphoma, NOS |
|  | Nodal MZL | 9699: Marginal zone B-cell lymphoma, NOS |
| **Follicular lymphoma** | Follicular lymphoma | 9690: Follicular lymphoma, NOS |
|  | Follicular lymphoma | 9691: Follicular lymphoma, grade 2 |
|  | Follicular lymphoma | 9695: Follicular lymphoma, grade 1 |
|  | Follicular lymphoma | 9698: Follicular lymphoma, grade 3 |
| **Hairy-cell leukemia** | Hairy-cell leukemia | 9940: Hairy cell leukemia |
| **Plasma cell neoplasms** | Plasma cell neoplasms | 9731: Plasmacytoma, NOS |
|  | Plasma cell neoplasms | 9732: Multiple myeloma |
|  | Plasma cell neoplasms | 9733: Plasma cell leukemia |
|  | Plasma cell neoplasms | 9734: Plasmacytoma, extramedullary |
| **Mycosis fungoides/Sezary syndrome** | Mycosis fungoides/Sezary syndrome | 9700: Mycosis fungoides |
|  | Mycosis fungoides/Sezary syndrome | 9701: Sezary syndrome |
| **Peripheral T-cell lymphoma** | Peripheral T-cell lymphoma | 9675*: ML, mixed sm. and lg. cell, diffuse |
|  | Peripheral T-cell lymphoma | 9702: Mature T-cell lymphoma, NOS |
|  | Peripheral T-cell lymphoma | 9724: SystemicEBV pos. T-cell lymphoproliferative disease of |
|  | Angioimmunoblastic T-cell lymphoma | 9705: Angioimmunoblastic T-cell lymphoma |
|  | Subcutaneous panniculitis-like T-cell lymph | 9708: Subcutaneous panniculitis-like T-cell lymphoma |
|  | Subcutaneous panniculitis-like T-cell lymph | 9726: Primary Cutaneous gamma-delta T-cell lymphoma |
|  | Anaplastic large cell lymphoma, T-/Null-cell | 9714: Anaplastic large cell lymphoma, T-cell and Null cell t |
|  | Hepatosplenic T-cell lymphoma | 9716: Hepatosplenic gamma-delta cell lymphoma |
|  | Enteropathy-type T-cell lymphoma | 9717: Intestinal T-cell lymphoma |
|  | Cutaneous T-cell lymphoma, NOS | 9709: Cutaneous T-cell lymphoma, NOS |
|  | Prim cutaneous anaplastic lar cell lymph | 9718: Primary cutan. CD30+ T-cell lymphoprolif. disorder |
| **Adult T-cell leukemia/lymphoma** | Adult T-cell leukemia/lymphoma | 9827: Adult T-cell leukemia/lymphoma (HTLV-1 pos.) |
| **NK/T-cell lymph, nasal-type/aggresive NK leuk** | NK/T-cell lymph, nasal-type/aggresive NK leuk | 9719: NK/T-cell lymphoma, nasal and nasal-type |
|  | NK/T-cell lymph, nasal-type/aggresive NK leuk | 9948: Aggressive NK-cell leukemia |
| **T-cell large granular lymphocytic leukemia** | T-cell large granular lymphocytic leukemia | 9831: T-cell large granular lymphocytic leukemia |
| **Composite** | Composite NHL/HL | 9596: Composite Hodgkin and non-Hodgkin lymphoma |
| **Non-Hodgkin lymphoma (NHL), not otherwise specified (NOS)** | NHL, NOS | 9591: Malignant lymphoma, non-Hodgkin |
|  | NHL, NOS | 9675*: ML, mixed sm. and lg. cell, diffuse |
|  | CNS lymphoma | 9590: Malignant lymphoma, NOS |
|  | CNS lymphoma | 9591: Malignant lymphoma, non-Hodgkin |
|  | CNS lymphoma | 9680: ML, large B-cell, diffuse |
|  | CNS lymphoma | 9684: ML, large B-cell, diffuse, immunoblastic, NOS |
| **Lymphoma, NOS** | Lymphoma, NOS | 9590: Malignant lymphoma, NOS |
|  | Lymphoma, NOS | 9971: Polymorphic PTLD |
| **Acute myelogenous leukemia (AML)** | AML | 9840: Acute myeloid leukemia, M6 type |
|  | AML | 9861: Acute myeloid leukemia |
|  | AML | 9865: Acute myeloid leukemia with t(6;9)(p23;q34) DEK-NUP214 |
|  | AML | 9867: Acute myelomonocytic leukemia |
|  | AML | 9869: Acute myeloid leukemia with inv(3)(q21q26.2) or t(3;3) |
|  | AML | 9871: Ac. myelomonocytic leuk. w abn. mar. eosinophils |
|  | AML | 9872: Acute myeloid leukemia, minimal differentiation |
|  | AML | 9873: Acute myeloid leukemia without maturation |
|  | AML | 9874: Acute myeloid leukemia with maturation |
|  | AML | 9891: Acute monocytic leukemia |
|  | AML | 9895: Acute myeloid leuk. with multilineage dysplasia |
|  | AML | 9896: Acute myeloid leukemia, t(8;21)(q22;q22) |
|  | AML | 9897: Acute myeloid leukemia, 11q23 abnormalities |
|  | AML | 9898: Myeloid leukemia associated with Down Syndrome |
|  | AML | 9910: Acute megakaryoblastic leukemia |
|  | AML | 9911: Acute myeloid leukemia (megakaryoblastic) with t(1;22) |
|  | AML | 9920: Therapy-related acute myeloid leukemia, NOS |
|  | AML | 9930: Myeloid sarcoma |
|  | AML | 9931: Acute panmyelosis with myelofibrosis |
|  | AML | 9987: Therapy-related myelodysplastic syndrome, NOS |
|  | APL | 9866: Acute promyelocytic leuk.,t(15;17)(q22;q11-12) |
|  | RAEBT | 9984: Refract. anemia with excess blasts in transformation |
| **Chronic myelogenous leukemia (CML)** | CML | 9863: Chronic myeloid leukemia, NOS |
|  | CML | 9875: Chronic myelogenous leukemia, BCR/ABL positive |
| **Leukemia, NOS** | Leukemia, NOS | 9800: Leukemia, NOS |
|  | Leukemia, NOS | 9801: Acute leukemia, NOS |
|  | Leukemia, NOS | 9820: Lymphoid leukemia, NOS |
|  | Leukemia, NOS | 9860: Myeloid leukemia, NOS |
| **Myelodysplastic syndrome (MDS)** | MDS | 9980: Refractory anemia |
|  | MDS | 9982: Refractory anemia with sideroblasts |
|  | MDS | 9983: Refractory anemia with excess blasts |
|  | MDS | 9985: Refractory cytopenia with multilineage dysplasia |
|  | MDS | 9986: Myelodysplastic syndr. with 5q deletion syndrome |
|  | MDS | 9989: Myelodysplastic syndrome, NOS |
| **Myelodysplastic/myeloproliferative disorders (MDS/MPN)** | MDS/MPN | 9975: Myelodysplastic/Myeloproliferative neoplasm, unclassif |
|  | JMML | 9946: Juvenile myelomonocytic leukemia |
|  | Atypical CML | 9876: Atypical chronic myeloid leuk., BCR/ABL negative |
|  | CMMoL | 9945: Chronic myelomonocytic leukemia, NOS |
| **Other myeloproliferative neoplasms (MPN)** | Other MPN | 9960: Chronic myeloproliferative disease, NOS |
|  | Other MPN | 9962: Essential thrombocythemia |
|  | Other MPN | 9964: Hypereosinophilic syndrome |
|  | PV | 9950: Polycythemia vera |
|  | PMF | 9961: Myelosclerosis with myeloid metaplasia |
| **Biphenotypic, bilineage or hybrid leukemia** | Biphenotypic, bilineage or hybrid leukemia | 9805: Acute biphenotypic leukemia |
|  | Biphenotypic, bilineage or hybrid leukemia | 9806: Mixed phenotype acute leukemia with t(9;22)(q34;q11.2) |
|  | Biphenotypic, bilineage or hybrid leukemia | 9807: Mixed phenotype acute leukemia with t(v;11q23);MLL rea |
|  | Biphenotypic, bilineage or hybrid leukemia | 9808: Mixed phenotype acute leukemia, B/myeloid, NOS |
|  | Biphenotypic, bilineage or hybrid leukemia | 9809: Mixed phenotype acute leukemia, T/myeloid, NOS |
| **Blastic plasmacytoid dendritic cell neoplasm (BPDCN)** | BPDCN | 9727: Precursor cell lymphoblastic lymphoma, NOS |
| * includes grade information to assign broad and detailed categories | | |
